# Levocetirizine Oral Disintegrating Tablet: A Randomized Open‐Label Crossover Bioequivalence Study in Healthy Japanese Volunteers

**DOI:** 10.1002/cpdd.791

**Published:** 2020-03-20

**Authors:** Hiroko Ino, Masanari Shiramoto, Takashi Eto, Miwa Haranaka, Shin Irie, Takumi Terao, Hirofumi Ogura, Akira Wakamatsu, Keiko Hoyano, Atsushi Nakano

**Affiliations:** ^1^ Clinical Pharmacology Office Japan Development Division GlaxoSmithKline KK Tokyo Japan; ^2^ Souseikai Hakata Clinic Fukuoka Japan; ^3^ Biomedical Data Sciences Department Japan Development Division GlaxoSmithKline KK Tokyo Japan; ^4^ Pre‐Clinical Development Department Japan Development Division GlaxoSmithKline KK Tokyo Japan; ^5^ Immuno‐Inflammation Therapeutic Office Medicines Development Japan Development GlaxoSmithKline KK Tokyo Japan

**Keywords:** Levocetirizine, cetirizine, oral disintegrating tablet, bioequivalence, healthy Japanese male subjects, single dose

## Abstract

Levocetirizine is classified as a second‐generation antihistamine. Levocetirizine is available for the treatment of allergic disorders such as allergic rhinitis and chronic idiopathic urticaria. This was a single‐center, single‐dose, open‐label, randomized, 2‐way crossover study in healthy Japanese male subjects consisting of 2 parts. Part 1 compared the bioavailability of levocetirizine oral disintegrating tablet (ODT) and levocetirizine immediate‐release tablet (IRT) taken with water in the fasted state in 24 subjects; all subjects completed this part of the trial. In part 2, the bioavailability of levocetirizine ODT without water was compared with that of levocetirizine IRT with water in the fasted state in 48 subjects; 47 subjects completed this part of the trial. Bioequivalence was demonstrated between levocetirizine IRT 5 mg and ODT 5 mg. The safety profiles were generally similar between levocetirizine ODT and levocetirizine IRT, with no serious adverse events, deaths, or adverse events leading to withdrawal reported during the study.

Levocetirizine hydrochloride (hereafter, levocetirizine), the R‐isomer of the racemic mixture cetirizine hydrochloride, is classified as a second‐generation antihistamine and is available for the treatment of allergic disorders, such as allergic rhinitis and chronic idiopathic urticaria.^1^ Levocetirizine has a high affinity and selective antagonistic activity against histamine H1 receptors and exerts an inhibitory effect on eosinophil chemotaxis.^2^, ^3^

Previous clinical studies and preclinical studies have shown that levocetirizine was rapidly and extensively absorbed.^1^, ^4^, ^5^ The time to reach the maximum concentration (t_max_) was between 0.8 and 1.0 hour after administration and decreased with a terminal half‐life (t_½_) of 7.3‐7.6 hours following the oral administration of levocetirizine 5 mg and 10 mg as a 5‐mg tablet formulation in healthy Japanese male subjects.^5^ The mean apparent volume of distribution of levocetirizine was low (0.3 L/kg), indicating that the distribution of levocetirizine is restrictive.^4^ Levocetirizine does not inhibit cytochrome P450 (CYP)1A2, 2C9, 2C19, 2D6, 2E1, and 3A4^1^ and does not induce uridine 5'‐diphospho‐glucuronosyltransferase 1A or CYP1A2, 2C9, or 3A4 at concentrations near the clinical dose, which produces peak plasma concentration (C_max_; in vitro study).^6^ The metabolic pathways involved in levocetirizine metabolism are oxidation (hydroxylation, O‐dealkylation, N‐oxidation, and N‐dealkylation), glucuroconjugation, taurine conjugation, and glutathione conjugation with formation of the mercapturic acids. Because levocetirizine is poorly metabolized and excreted primarily as unchanged drug (77% in urine and 9% in feces), concomitant medications are unlikely to significantly alter the pharmacokinetics (PK) of levocetirizine.^4^

Orally disintegrating tablets (ODTs) are used widely in drug therapy and are clinically attractive because they are suitable for use in patients with dysphagia, improve adherence, increase the likelihood of achieving the desired therapeutic effect, and are convenient to take without water.^7^ Levocetirizine is currently available as a 5‐mg oral tablet (conventional immediate‐release tablet [IRT]) and 0.05% oral solution. A new dosage form of levocetirizine, an ODT, is under development for the purpose of improving convenience and compliance for patients with allergic diseases.

This study was designed to assess the bioequivalence (BE) of a single oral dose of 5 mg levocetirizine administered as an ODT and an IRT in healthy Japanese male subjects.

## Methods

### Study Design and Study Population

The study was conducted between May 18, 2018 and September 17, 2018 at Souseikai Hakata Clinic in Japan, in accordance with the International Conference on Harmonisation Good Clinical Practice guidelines, all applicable participant privacy requirements, and the ethical principles outlined in the current version of the Declaration of Helsinki. The study protocol and informed consent documents were approved by the Institutional Review Board of the clinic. Written informed consent was obtained from each participant before any screening evaluations.

This study consisted of 2 parts: part 1 compared the bioavailability of levocetirizine ODT and levocetirizine IRT taken with water in the fasted state, and part 2 compared the bioavailability of levocetirizine ODT without water and levocetirizine IRT with water in the fasted state (Clinicaltrials.gov identifier: NCT03555890).

BE between levocetirizine ODT and levocetirizine IRT was evaluated in 48 healthy Japanese male subjects (part 1, 24 subjects; part 2, 24 subjects) in the first study (hereafter, the “initial study”). Because BE was not demonstrated in part 2 of the initial study, an add‐on subject study was conducted in 24 subjects using the same methodology as used in the initial study of part 2 in accordance with the Japanese Guideline for Bioequivalence Studies of Generic Products issued by Evaluation and Licensing Division, Pharmaceutical and Food Safety Bureau, Ministry of Health, Labour, and Welfare (hereafter Japanese BE guidelines).

In each part (part 1 and part 2 of the initial study, and the add‐on subject study), subjects underwent a screening visit within 30 days before the first dose of study drug, participated in a 2‐period 2‐way crossover intervention with at least a 5‐day washout period between interventions, and took part in a follow‐up visit 5‐7 days after the final dose. All subjects stayed in the clinical research unit from day –1 (the day before dosing) until 48 hours postdose for each assessment period.

In each part, 24 subjects were divided into 2 equal groups (12 subjects in each group) and were randomized in a 1:1 ratio to 1 of 2 groups in each part. Subjects participated only in either part 1 or part 2 (initial study or add‐on subject study, respectively). All subjects received a single dose of levocetirizine ODT or IRT after an overnight fast (at least 10 hours) during the intervention period.

The key inclusion criteria were age 20‐55 years, body mass index of 18.5‐24.9 kg/m^2^, and good general health as determined by medical history, clinical examination, 12‐lead ECG, and clinical laboratory tests.

### Pharmacokinetic Sample Collection and Bioanalytical Methods

Blood samples for the determination of levocetirizine concentration in plasma were collected before dosing and at the following nominal times relative to the time of levocetirizine administration after dosing in each part: 0.25, 0.5, 1, 1.5, 2, 3, 4, 6, 9, 12, 16, 24, 36, and 48 hours.

Two‐milliliter blood samples were collected into a K_2_‐EDTA tube, which was immediately inverted 10 times and placed on ice‐cold water until centrifugation. The samples were centrifuged at 1500*g* for 10 minutes at 4°C within 1 hour of blood collection. The supernatant plasma was transferred to a 1.8‐mL polypropylene tube and stored at –20°C or below until shipment. The samples were shipped frozen with dry ice according to the schedule throughout the study to the designated bioanalytical facility, Shin Nippon Biomedical Laboratories (Wakayama, Japan).

Plasma levocetirizine concentration was analyzed using an analytical method, validated by Shin Nippon Biomedical Laboratories based on a previously reported method^8^ with slight modification: less plasma volume and different assay range, which was based on liquid‐liquid extraction, followed by high‐performance liquid chromatography‐tandem mass spectrometry (HPLC‐MS/MS). Briefly, levocetirizine was extracted by liquid‐liquid extraction after protein precipitation of 100 µL of human plasma containing an isotopically labeled internal standard (cetirizine‐d_8_ dihydrochloride). The extracts were analyzed by using an HPLC‐MS/MS system (API4000; Sciex, Framingham, Massachusetts) with an atmospheric pressure chemical ionization interface in the positive‐ion mode. Multiple reaction monitoring was used; the transition masses for levocetirizine and the internal standard were 389.1 to 201.3 and 397.3 to 201.3, respectively. The lower limit of quantification was 2 ng/mL for a 100‐µL aliquot of EDTA plasma. The upper limit of quantification was 1000 ng/mL. The intra‐ and interassay bias and precision for levocetirizine were less than 15% with the exception of the lower limit of quantification, for which less than 20% was acceptable.

### Safety Assessments

Safety parameters included adverse events (AEs), clinical laboratory tests (hematology, chemistry, urinalysis), vital signs (blood pressure, pulse rate, and body temperature), and 12‐lead ECGs, which were monitored throughout the study.

### PK and Statistical Analyses

The PK parameters for levocetirizine were calculated from the plasma levocetirizine concentrations and actual sampling times by standard noncompartmental methods using Phoenix WinNonlin version 7.0 (Certara USA, Princeton, New Jersey). The following levocetirizine PK parameters were determined: C_max_, area under the curve (AUC_0‐t_), AUC_0‐inf_, t_max_, and t_½_.

For both part 1 and part 2, after log_e_ transformation, AUC_0‐t_ and C_max_ of levocetirizine 5 mg, when given as ODT or IRT, were analyzed separately using mixed‐effects model fitting for intervention and period as fixed effects and subject as random effect. The Kenward‐Roger degrees of freedom approximation was used. Point estimates and associated 90% CIs for the difference in means of 2 interventions (logμ_test_ – logμ_ref_) were constructed by using the residual variance. These estimated values were exponentially back‐transformed to provide point estimates and associated 90% CIs for the geometric mean ratio (μ_test_/μ_ref_).

In both parts the interventions were considered to be bioequivalent if the 90% CIs of the geometric mean ratio of AUC_0‐t_ and C_max_ were within the acceptable range (0.80‐1.25). If BE was not demonstrated in part 1 and/or part 2 because of an insufficient number of subjects, an add‐on subject study was planned to have been performed with 12 (6 subjects in each group) or more subjects per part using the same methodology and according to the Japanese BE guidelines. In the case in which the add‐on subject study was conducted, the data from the 2 studies (initial study and add‐on subject study) were combined for the analysis, and the term of study (initial study or add‐on subject study) was included in the analysis model as fixed effect. When PK parameter data were missing for a subject in period 1 or period 2, the subject data were analyzed by using the mixed‐effects model to evaluate the bioequivalence of PK parameter.

The sample size calculation was based on the within‐subject estimates of variability (CVw%). CVw% values of approximately 6% and 11% for AUC_0‐48_ and C_max_ had been observed, respectively, in a previous study of healthy Japanese male subjects.^9^ Based on the conservative assumption for CVw% of 22% (double 11%), it was estimated that a sample size of 23 subjects provided a power of at least 90% to demonstrate bioequivalence in each part. After consideration of the treatment assignment, a total of 24 healthy Japanese male subjects were enrolled in each part of the study. This calculation was based on 2‐sided t‐test procedure with a type 1 error rate of 5% and an assumed a true ratio of 1.00. This procedure corresponded to the acceptance criteria for 90% CI.

The safety population was defined as all participants who received at least 1 dose of study medication. The PK population was defined as all participants from whom a PK sample was obtained and analyzed. The demographic characteristics, safety, and palatability were analyzed for the safety population. The PK analyses were performed on the PK population.

BE was not demonstrated in part 2 of the initial study in 24 subjects: although the 90% CI of the AUC_0‐t_ met the BE criteria, the 90% CI of the C_max_ did not meet the BE criteria (Table 1). To potentially meet the BE criteria through the addition of further subjects, an add‐on subject study with the same design as part 2 was performed. An additional 24 subjects (12 subjects in each group) was studied to increase the number of subjects to be included in the BE assessment. In part 2 the data from both studies (initial study and add‐on subject study) were combined for analysis.

**Table 1:** Summary of Bioequivalence Analysis on AUC_0‐t_ and C_max_

	Geometric LS Mean				
Parameter	Levocetirizine ODT 5 mg	Levocetirizine IRT 5 mg^a^	Ratio^b^	90% CI of the Ratio	%CVw
Part 1, levocetirizine ODT 5 mg administered with water (N = 24)					
AUC_0‐t_ (h∙ng/mL)	1758.5	1803.2	0.975	0.948‐1.003	5.6
C_max_ (ng/mL)	219.9	235.4	0.934	0.875‐0.998	13.3
Part 2 (initial study), levocetirizine ODT 5 mg administered without water (N = 24)					
AUC_0‐t_ (h∙ng/mL)	1712.9	1776.6^a^	0.964	0.942‐0.987	4.5
C_max_ (ng/mL)	198.6	239.9^a^	0.828	0.762‐0.900	16.6
Part 2 (initial + add‐on subject studies, levocetirizine ODT 5 mg administered without water (N = 48)					
AUC_0‐t_ (h∙ng/mL)	1809.9	1850.7^b^	0.978	0.958‐0.998	5.9
C_max_ (ng/mL)	191.5	223.4^b^	0.857	0.815‐0.902	14.6

AUC_0‐t_ indicates area under the concentration‐time curve, 0 to the last time of quantifiable concentration; C_max_, maximum observed concentration; CVw(%), coefficient of variation within subjects; IRT, immediate‐release tablet; LS Mean, least‐squares mean; N, number of subjects analyzed; n, number of subjects with available data (*n=23, **n=47); ODT, oral disintegrating tablet.

a Administered with water.

b Ratio calculated as geometric least‐squares mean of levocetirizine ODT 5 mg/geometric least squares mean of levocetirizine IRT 5 mg.

SAS version 9.4 (SAS Institute, Cary, North Carolina) was used to create data sets and perform the statistical analyses.

## Safety Analyses

Adverse events, clinical laboratory tests, vital signs, and 12‐lead ECGs were summarized by treatment in each part. AEs were coded with the *Medical Dictionary for Regulatory Activities*, *version 21.0*.

## Palatability Assessment

A palatability questionnaire was administered to each subject within 10 minutes after dosing of the ODT treatment. The Palatability Questionnaire is provided in Supplement 1. Subjects were given the questionnaire to read before receiving this dose. The palatability of the product was rated by each subject as unacceptable, neutral/acceptable, or very good.

## Results

### Participant Disposition and Demographics

The demographic characteristics of subjects in part 1 and part 2 are summarized in Table 2.

**Table 2:** Summary of Demographic Characteristics

Demographics	Part 1 (N = 24)	Part 2 (Initial^a^) (N = 24)	Part 2 (Initial + Add‐on^b^) (N = 48)
Age (y)	30.6 (7.81)	28.8 (6.60)	26.7 (6.68)
BMI (kg/m^2^)	21.45 (2.019)	21.28 (2.135)	21.31 (1.964)
Height (cm)	171.10 (6.143)	172.91 (5.501)	172.56 (5.395)
Weight (kg)	62.97 (8.315)	63.71 (7.695)	63.50 (6.827)

BMI indicates body mass index; N, number of subjects.

Values are arithmetic mean (SD).

a Initial indicates the initial study.

Initial + Add‐on indicates the initial study and add‐on subject study.

### Part 1: Administration of Levocetirizine ODT With Water

Of the 51 healthy Japanese male subjects screened, 24 subjects were randomized and completed the study.

### Part 2: Administration of Levocetirizine ODT Without Water

Of the 38 and 44 healthy Japanese male subjects screened in the initial study and add‐on subject study, respectively, 24 subjects each were randomized and received at least 1 dose of the investigational products. All subjects except 1 who were randomized in the initial study completed the study. One subject was withdrawn from the initial study after receiving the ODT dose in period 1. He experienced a moderate AE (tonsillitis) during the washout period, and his dosing schedule for period 2 was postponed; however, he withdrew his consent because his schedule did not suit the new study schedule for the in‐clinic stay.

## PK and BE of Oral Levocetirizine Formulations

### Part 1: Administration of Levocetirizine ODT With Water

**Figure 1:**
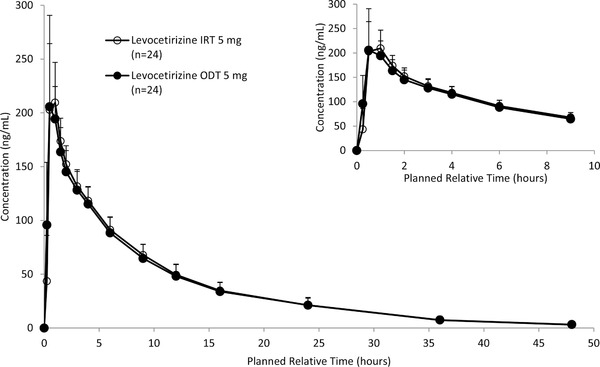
Mean (+SD) levocetirizine plasma concentrations over time for the 2 formulations after dosing of ODT 5 mg with water and IRT 5 mg with water (part 1). The lower limit of quantification is 2 ng/mL. IRT indicates immediate‐release tablet; n, number of subjects with available data; ODT, oral disintegrating tablet.

The mean levocetirizine plasma concentrations over time for the 2 formulations after dosing of ODT with water and IRT with water are shown in Figure 1. The t_max_ of levocetirizine was 0.50 hour and 0.75 hour, respectively, and plasma concentrations subsequently declined with a t_½_ of 8.92 hours and 8.66 hours, respectively (Table 3). After dosing of each formulation, 17 and 18 out of 24 subjects had quantifiable concentrations of levocetirizine (> 2.0 ng/mL) for up to 48 hours postdose, respectively. The arithmetic mean values of AUC_0‐t_ were 1791.4 h∙ng/mL and 1827.6 h∙ng/mL, respectively, and those of C_max_ were 224.0 ng/mL and 240.9 ng/mL, respectively. The point estimate (90% CI) of the ODT/IRT for AUC_0‐t_ was 0.975 (0.948‐1.003). Similarly, the 90% CI of the ODT/IRT for C_max_ was 0.934 (0.875‐0.998) (Table 1). The 90% CI for in AUC_0‐t_ and C_max_ was within the BE criteria of 0.80‐1.25.

**Table 3:** Summary of Levocetirizine Plasma PK Parameters Following a Single‐Dose Administration

Treatment	N	C_max_ (ng/mL)	AUC_0‐t_ (h*ng/mL)	AUC_0‐inf_ (h*ng/mL)	t_max_ (h)	t_½_ (h)
Part 1						
Levocetirizine ODT 5 mg with water	24	224.0 (43.07)	1791.4 (344.41)	1848.7 (366.84)	0.50 (0.50, 1.50)	8.92 (1.305)
Levocetirizine IRT 5 mg with water	24	240.9 (51.07)	1827.6 (300.05)	1879.2 (318.89)	0.75 (0.50, 1.50)	8.66 (1.460)
Part 2 (initial study)						
Levocetirizine ODT 5 mg without water	24	202.3 (38.81)	1750.4 (369.49)	1807.8 (392.94)	1.00 (0.50, 3.00)	8.75 (1.522)
Levocetirizine IRT 5 mg with water	23	247.1 (59.82)	1795.3 (377.51)	1847.8 (400.49)	1.00 (0.50, 1.50)	8.68 (1.337)
Part 2 (initial + add‐on subject studies)						
Levocetirizine ODT 5 mg without water	48	194.6 (34.96)	1839.2 (325.32)	1902.3 (351.96)	1.00 (0.50, 4.00)	9.14 (1.451)
Levocetirizine IRT 5 mg with water	47	230.6 (60.11)	1872.7 (334.00)	1933.1 (362.93)	1.00 (0.50, 3.00)	9.03 (1.388)

AUC_0‐t_ indicates area under the concentration‐time curve, 0 to the last time of quantifiable concentration; AUC_0‐inf_, area under the concentration‐time curve from time 0 to infinity; C_max_, maximum observed concentration; IRT, Immediate release tablet; N, number of subjects with available data; ODT, oral disintegrating tablet; t_½_, terminal half‐life; t_max_, time to maximum observed concentration.

Values are arithmetic mean (SD) except for t_max_, which is median (min, max).

### Part 2: Administration of Levocetirizine ODT Without Water, Initial Study

**Figure 2:**
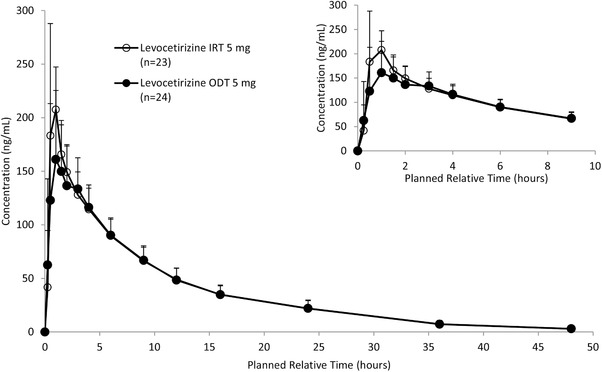
Mean (+SD) levocetirizine plasma concentrations over time for the 2 formulations after dosing of ODT 5 mg without water or IRT 5 mg with water (part 2, initial study). The lower limit of quantification is 2 ng/mL. IRT indicates immediate‐release tablet; n, number of subjects with available data; ODT, oral disintegrating tablet.

The mean levocetirizine plasma concentrations over time for the 2 formulations after dosing of ODT 5 mg without water or IRT 5 mg with water are shown in Figure 2. The t_max_ of levocetirizine was 1.00 hour in both cases, and plasma concentrations subsequently declined with a t_½_ of 8.75 hours and 8.68 hours, respectively (Table 3). After dosing of ODT without water and IRT with water, 16 and 17 out of 24 subjects had quantifiable concentrations of levocetirizine (>2.0 ng/mL) up to 48 hours postdose, respectively. The arithmetic mean values of AUC_0‐t_ were 1750.4 h∙ng/mL and 1795.33 h∙ng/mL, respectively, and those of C_max_ were 202.3 ng/mL and 247.1 ng/mL, respectively.

The point estimates (90% CI) of the ODT/IRT for AUC_0‐t_ and C_max_ were 0.964 (0.942‐0.987) and 0.828 (0.762‐0.900), respectively, which did not meet the BE criteria (Table 1). To potentially meet the BE criteria by the addition of more subjects, an add‐on subject study with the same design as part 2 was performed. An additional 24 subjects (12 subjects in each group) were studied to increase the number of subjects included in the BE assessment. The additional sample size was considered with a significance level of 5% (1‐sided) and a power of 90% when the minimum ratio of geometric mean was set at 0.9. If CVw% was set as 19% which is the mean of the result and the plan of the study, 22 additional subjects were required. Considering the feasibility of conducting additional study, 24 additional subjects were studied, which was same sample size as the initial study.

### Part 2: Administration of Levocetirizine ODT Without Water, Initial Study and Add‐on Subject Study

**Figure 3:**
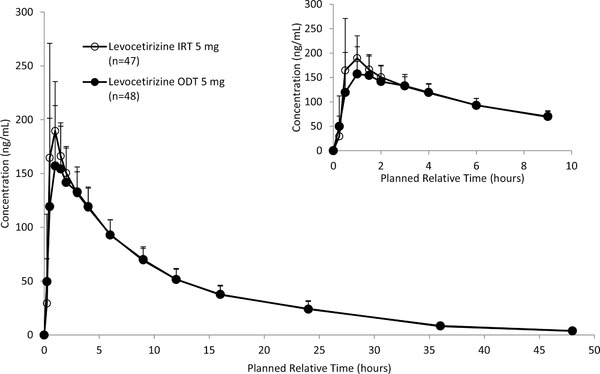
The combined (initial study and add‐on subject study) results of the mean (+SD) levocetirizine plasma concentrations over time for the 2 formulations after dosing of ODT 5 mg without water or IRT 5 mg with water (part 2, initial and add‐on subject study). The lower limit of quantification is 2 ng/mL. IRT indicates immediate‐release table; n, number of subjects with available data; ODT, oral disintegrating tablet.

The combined (initial study and add‐on subject study) results of the mean levocetirizine plasma concentrations over time for the 2 formulations after dosing of ODT 5 mg without water or IRT 5 mg with water are shown in Figure 3. The t_max_ of levocetirizine was 1.0 hour, and the plasma concentrations subsequently declined with a t_½_ of 9.14 hours and 9.03 hours, respectively (Table 3). Thirty‐nine subjects in each formulation group had quantifiable concentrations of levocetirizine (> 2.0 ng/mL) for up to 48 hours postdose.

Following a single oral dose of levocetirizine ODT 5 mg without water and levocetirizine IRT 5 mg with water in the fasted state, the arithmetic mean values of AUC_0‐t_ were 1839.2 h∙ng/mL and 1872.7 h∙ng/mL, respectively, and those of C_max_ were 194.6 ng/mL and 230.6 ng/mL, respectively (Table 3).

The point estimates (90% CI) of the ODT/IRT for AUC_0‐t_ and C_max_ were 0.978 (0.958‐0.998) and 0.857 (0.815‐0.902), respectively (Table 1). The 90% CIs for AUC_0‐t_ and C_max_ were within the BE criteria of 0.80‐1.25 after the combined analyses of the initial and add‐on subject studies.

## Safety

### Part 1: Administration of Levocetirizine ODT With Water

No subject reported an AE in part 1.

### Part 2: Administration of Levocetirizine ODT Without Water

In the initial and add‐on subject studies, 1 (2%) out of 48 subjects who received levocetirizine ODT 5 mg experienced drug‐related AEs (2 mild AEs: alanine transaminase increased, and aspartate transaminase increased), although no AEs were reported in 47 subjects who received levocetirizine IRT 5 mg. The increases in alanine transaminase and aspartate transaminase were transient and had returned to the normal range by the end of the study without treatment. There were no clinically significant findings in clinical laboratory evaluations in the other subjects in the study. There were no safety concerns with respect to vital signs (blood pressure, pulse rate, and body temperature) or ECG after the administration of both products.

## Palatability

The results of the palatability evaluation for part 1 and part 2 are summarized in Table 4.

**Table 4:** Summary of Palatability Questionnaire

Parameter	Acceptability of Taste	Levocetirizine ODT 5 mg With Water (N = 24)	Levocetirizine ODT 5 mg Without Water (N = 48)
Palatability rate, n (%)	Unacceptable	0	1 (2)
	Neutral/acceptable	16 (67)	39 (81)
	Very good	8 (33)	8 (17)

N indicates number of subjects; n, number of subjects for each rating category of acceptability of taste; ODT, oral disintegrating tablet.

The questionnaire was administered to each subject within 10 minutes following a 5‐mg dose of the levocetirizine where it had been given as an ODT. Subject was given the questionnaire to read before receiving this dose.

### Part 1: Administration of Levocetirizine ODT With Water

Sixteen subjects (67%) rated the palatability of the product as neutral/acceptable, and 8 subjects (33%) rated the palatability as very good. No subject rated the palatability as unacceptable.

### Part 2: Administration of Levocetirizine ODT Without Water

Thirty‐nine subjects (81%) rated the palatability of the product as neutral/acceptable, and 8 subjects (17%) rated the palatability as very good. One subject (2%) rated the palatability as unacceptable.

## Discussion

This study was conducted to determine the BE between levocetirizine ODT 5 mg (test product) and levocetirizine IRT 5 mg (reference product) because a new dosage form of levocetirizine, an ODT, has been developed with the aim of improving the convenience and compliance of patients with allergic disease.

The observed exposures (AUC_0‐t_ and C_max_) were slightly higher after the administration of levocetirizine IRT 5 mg with water than after the administration of levocetirizine ODT 5 mg with water (part 1) and without water (part 2). Although the 90%CI for the geometric mean ratios of AUC_0‐t_ and C_max_ for levocetirizine ODT 5 mg administered with water, compared with levocetirizine IRT 5 mg with water, was within the accepted BE range, the BE criteria were not met for levocetirizine ODT 5 mg administered without water compared with levocetirizine IRT 5 mg with water in the 24 subjects who participated in part 2 of the initial study. The ratios of the geometric means for the AUC_0‐t_ and C_max_ of levocetirizine ODT 5 mg without water to levocetirizine IRT 5 mg with water were 0.964 and 0.828, and the 90%CIs were 0.942‐0.987 and 0.762‐0.900, respectively. For C_max_, the %CVw (16.6%) was smaller than the value (22%) expected at study planning, whereas the ratio of 0.828 was different from unity, which was assumed during study planning. Therefore, this may explain why BE was not observed in the initial study of part 2.

In accordance with the BE guideline, the add‐on subject study was conducted with the same methodology as the initial study, and with an additional 24 subjects, only for the "without water” condition. When the data from the initial study and add‐on subject study were combined for analysis, the 90%CIs of the geometric mean ratio of AUC_0‐t_ and C_max_ were within the accepted bioequivalence range of 0.80‐1.25. Overall, BE was demonstrated between levocetirizine ODT 5 mg and levocetirizine IRT 5 mg.

The safety profiles were generally similar between levocetirizine ODT 5 mg and levocetirizine IRT 5 mg, which indicates that both treatments were well tolerated. In addition, more than 98% of subjects rated the palatability of the product as neutral/acceptable or very good after the administration of ODT without water. This finding supports the idea that ODT may be taken without water.

This study confirmed that levocetirizine ODT 5 mg was bioequivalent to levocetirizine IRT 5 mg, supporting the replacement of 5 mg levocetirizine tablets with 5 mg levocetirizine ODT at the clinical site. Because these tablets dissolve directly in the mouth, their taste is also an important factor.^7^ The palatability of levocetirizine ODT was rated as neutral/acceptable or very good in more than 98% of subjects. Therefore, ODTs should help achieve the proper peroral administration of levocetirizine, especially in pediatric and geriatric populations, where swallowing may hinder compliance.

## Conclusions

Bioequivalence was demonstrated between levocetirizine IRT 5 mg and ODT 5 mg. The safety and tolerability of levocetirizine were confirmed following single oral administrations of levocetirizine IRT 5 mg and ODT 5 mg administered with or without water. Thus, a 5 mg levocetirizine ODT tablet can be used as a replacement for a 5 mg levocetirizine IR tablet at the clinical site.

## Funding

GlaxoSmithKline sponsored and provided funding for the study as well as for third‐party editorial support to Mediscience Planning Inc.

## Disclosures

As the study was being conducted, 4 authors including M.S., T.E., M.H., and S.I. were employees of SOUSEIKAI Hakata Clinic, and H.I., T.T., H.O., A.W., K.H., and A.N. were employees of GlaxoSmithKline.

## Supporting information

Supporting Information
