# Supplementary material for: Levocetirizine Oral Disintegrating Tablet: A Randomized Open‐Label Crossover Bioequivalence Study in Healthy Japanese Volunteers
Source: Clin Pharmacol Drug Dev. 2020 Mar 20;9(7):805–12. doi: 10.1002/cpdd.791 (PMC7586835; doi:10.1002/cpdd.791)
Supplement: Supplementary file 1 — Supporting Information [file CPDD-9-805-s001.docx]

**Palatability Questionnaire**

1. Please briefly describe the taste of the product in your own words (one word, short phrase descriptions are acceptable).
2. Please rate the palatability (acceptability of taste) of the product by checking a rating below.

____ 1 = unacceptable (would not use product under any circumstances)
____ 2 = neutral/acceptable
____ 3 = very good

1. Please check **all** the descriptors that apply to the product.

______ Sweet

______ Sour/tart

______ Bitter

______ Fruity

______ Nutty

______ Chalky

______ Medicinal

1. Please rate the mouth feel of the product by checking a rating below.

_____ 1 = unacceptable (would not use product under any circumstances)
_____ 2 = neutral/acceptable
_____ 3 = very good

1. For each of the following attributes please circle the number that best describes your perception of each attribute.
2. Sweetness

Not Sweet Sweet Very Sweet

1. Sour/tartness

Mild Average Strong

1. Bitterness

Not bitter Bitter Very Bitter
